# Gene expression profiles associated with acute myocardial infarction and risk of cardiovascular death

**DOI:** 10.1186/gm560

**Published:** 2014-05-30

**Authors:** Jinhee Kim, Nima Ghasemzadeh, Danny J Eapen, Neo Christopher Chung, John D Storey, Arshed A Quyyumi, Greg Gibson

**Affiliations:** 1 Center for Integrative Genomics and School of Biology, Georgia Institute of Technology, Boggs Building 1-96, 770 State Street, Atlanta, GA 30332, USA; 2 School of Medicine, Division of Cardiology, Emory University, Atlanta, GA 30322, USA; 3 Lewis-Sigler Institute, Carl Icahn Labs, Princeton University, Princeton, NJ 08544, USA

## Abstract

**Background:**

Genetic risk scores have been developed for coronary artery disease and atherosclerosis, but are not predictive of adverse cardiovascular events. We asked whether peripheral blood expression profiles may be predictive of acute myocardial infarction (AMI) and/or cardiovascular death.

**Methods:**

Peripheral blood samples from 338 subjects aged 62 ± 11 years with coronary artery disease (CAD) were analyzed in two phases (discovery N = 175, and replication N = 163), and followed for a mean 2.4 years for cardiovascular death. Gene expression was measured on Illumina HT-12 microarrays with two different normalization procedures to control technical and biological covariates. Whole genome genotyping was used to support comparative genome-wide association studies of gene expression. Analysis of variance was combined with receiver operating curve and survival analysis to define a transcriptional signature of cardiovascular death.

**Results:**

In both phases, there was significant differential expression between healthy and AMI groups with overall down-regulation of genes involved in T-lymphocyte signaling and up-regulation of inflammatory genes. Expression quantitative trait loci analysis provided evidence for altered local genetic regulation of transcript abundance in AMI samples. On follow-up there were 31 cardiovascular deaths. A principal component (PC1) score capturing covariance of 238 genes that were differentially expressed between deceased and survivors in the discovery phase significantly predicted risk of cardiovascular death in the replication and combined samples (hazard ratio = 8.5, *P* < 0.0001) and improved the C-statistic (area under the curve 0.82 to 0.91, *P* = 0.03) after adjustment for traditional covariates.

**Conclusions:**

A specific blood gene expression profile is associated with a significant risk of death in Caucasian subjects with CAD. This comprises a subset of transcripts that are also altered in expression during acute myocardial infarction.

## Background

Coronary artery disease (CAD) is a complex multifactorial disease resulting from the interplay between genetic, environmental, and behavioral factors. The promise of genetic research in CAD includes development of unique genomic risk scores to enhance risk prediction and discovery of novel therapeutic targets [1-3]. Although genome-wide association studies have identified numerous SNPs that associate with CAD [4-7], as with most complex traits, the discovered variants explain only a small fraction of risk [8]. Recent advances in gene expression analysis have also discovered transcriptomic signatures in peripheral blood that associate with presence and severity of CAD as well as risk of adverse events, possibly in a sex-specific manner [9,10].

Numerous studies have combined SNPs identified in genome-wide association studies to generate risk scores for CAD, in some cases generating marginal improvement on traditional risk models [1,11,12]. There is also a pressing need for biomarkers of risk of cardiac events within the incident CAD patient population. One small study of 28 cases [13] documented a peripheral blood gene expression signature associated with acute myocardial infarction (AMI) that persisted to some extent on follow-up. It is not clear whether this signature is broadly predictive of risk or simply represents perturbation during the event. Addressing this distinction requires a large prospective cohort with RNA samples archived for follow-up assessment of adverse cardiovascular events. Here, we performed Illumina microarray-based gene expression profiling to quantify the relative abundance of transcripts in peripheral blood that are associated with AMI and/or CAD. Further, we investigated whether these markers are also associated prospectively with the risk of future cardiovascular death. We show that two axes of gene expression co-variation, each including hundreds of transcripts related to lymphocyte and neutrophil activity, are differentially expressed during AMI. A distinct subset of these generate a predictor of future cardiovascular death that replicates in two phases of analysis and is strongly associated with survival time in the combined dataset.

## Methods

### Subjects

We studied 338 subjects with suspected or confirmed CAD undergoing cardiac catheterization who were enrolled in the Emory Cardiovascular Biobank. The study was approved by the institutional review boards at Emory University and the Georgia Institute of Technology, Atlanta, GA, USA, and was conducted in accordance with the principles of the Declaration of Helsinki. All subjects provided written informed consent. Subjects were evaluated in two phases, the discovery phase with 175 subjects and replication phase with 163 subjects. Coronary angiograms were scored for luminal narrowing using a modified American Heart Association/American College of Cardiologists classification [14]. Patients were designated as having non-significant CAD (visible plaque resulting in <50% luminal stenosis), or significant CAD (at least one major epicardial vessel with ≥50% stenosis). Subjects with congenital heart disease, severe valvular heart disease, history of orthotopic heart transplant, severe anemia, recent blood transfusion, active inflammatory diseases, or cancer were excluded. Clinical characteristics and behavioral factors were obtained using a comprehensive questionnaire and were confirmed by electronic chart review. Risk factor prevalence was determined by physician diagnosis and/or treatment of hypertension, hyperlipidemia, and diabetes and detailed medication history was obtained [15]. Prevalent myocardial infarction (MI) at the time of enrollment was diagnosed using standard universal criteria. Quantitative angiographic scoring was performed using the Gensini score that quantifies CAD severity by a nonlinear points system for degree of luminal narrowing. The score has prognostic significance [16].

### Clinical outcome measures

Based on their initial evaluation, patients were divided into those with non-significant CAD, those with significant CAD by the above criteria, those with AMI on admission, and those with a history of MI (labeled NO CAD, CAD, AMI and OLD MI, respectively). Follow-up evaluation was conducted by telephone interviews and by chart review by personnel blinded to the gene expression data, between 1 and 5 years (mean 2.4 years) after blood sampling, in order to determine all cause death, non-fatal MI, and cardiovascular death. Records were also linked to the Social Security Death Index and State of Georgia records. Cardiovascular death was defined as death attributable to an ischemic cardiovascular cause (fatal MI, stroke, peripheral arterial disease) or sudden death due to an unknown cause [15]. Medical records were accessed or requested to validate all self-reported events including MI, which was defined using standard international criteria for diagnosis.

### Gene expression profiling

Gene expression data have been deposited at the Gene Expression Omnibus archive under accession number [GEO:GSE49925]. Peripheral blood samples were collected immediately prior to angiography and after overnight fasting, and stored in Paxgene tubes (QIAGEN, San Diego, CA, USA) at -80°C. Microarray analysis of transcript abundance was performed by hybridization of dye-labeled RNA to Illumina HT-12 bead arrays containing probes for all human reference genes. Hybridizations for the discovery phase were performed by Expression Analysis (Durham, NC, USA) and for the replication phase by HudsonAlpha (Huntsville, AL, USA). Average bead intensity values were exported from the Illumina GenomeStudio, log base 2 transformed, and 14,343 probes that are consistently detected above background in multiple gene expression datasets that we have analyzed [17-19] were retained for subsequent analyses. Of these probes, 232 were missing in the replication phase analysis using an updated version of the Illumina HT-12 arrays, resulting in 14,111 probes analyzed in both phases.

Two modes of data normalization were pursued in order to confirm robustness of all conclusions [20]. The primary analysis method reported in the main text employed the Supervised Normalization of Microarray (SNM) algorithm [21,22]. Secondary analyses were performed by linear mixed modeling at the transcript level [23]. All downstream analysis of variance and regressions on traits was performed with JMP Genomics v5 (SAS Institute, Cary, NC, USA). Normalization was initially performed independently on the two phases to ensure independent replication, and subsequently the data were combined for meta-analysis. The variance components attributable to body mass index (BMI), gender, ethnicity, CAD status and technical plate effects are shown in Additional file 1.

SNM was implemented in two steps in order to remove technical effects and adjust for biological covariates. In step one, we fit CAD status (with four levels: NO CAD, CAD, AMI and OLD MI) as the biological variable of interest, and adjusted for BMI, gender, ethnicity (in the discovery phase only since all individuals in the replication phase were Caucasian) and age (in the replication phase only, since it was not correlated with the major components of variance in the discovery phase). In step two, we also fit CAD status as the biological variable, but removed the technical effects of RNA quality (bioanalyzed RNA integrity number (RIN) number) and plate (two per phase) using the rm.adj = TRUE option. The π_0_ estimate for both phases was between 60 and 70%, indicating that this proportion of the transcripts was unaffected by presence or absence of significant CAD status but suggesting that up to as many as 4,500 transcripts may differ between those with and without CAD or AMI. Code and input. csv files are available from the authors’ website [24].

For the linear regression normalization, log2 fluorescence intensity measures were mean-centered by individual and linear regression fitting RNA quality and plate was performed, after which the residuals for each probe were converted to standardized normal distributions (z-scores). These were then re-centered by individual by subtracting the mean z-score of all probes, yielding the normalized gene expression measures.

### Expression quantitative trait locus analysis

Whole genome genotypes for the discovery phase were determined by Illumina OmniQuad arrays at Expression Analysis (Durham, NC, USA), and retained where calls were made for 95% of the individuals and Hardy-Weinberg equilibrium observed at *P* > 0.001. Expression quantitative trait locus (eQTL) analysis was performed only on the Caucasian samples (n = 153) by linear regression against the SNM normalized expression values (similar results were observed for the alternative normalization). Genotypes were excluded if the minor allele frequency was less than 0.1, to avoid rare homozygotes biasing the analysis, and we also required at least two individuals in each genotype class. Only local (*cis*) associations (SNPs within 250 kb of the probe) were assessed since this analysis is underpowered to assess distal (*trans*) associations, and in Additional file 2 we report only the strongest association per locus since the study is underpowered to detect multiple associations even in the presence of reduced linkage disequilibrium. In addition, 3,651 probes known to contain common SNPs were removed from the analysis [25], including 39 putativeeQTL.

### Principal component analysis and axes of variation

Principal component analysis (PCA) was used to define the major orthogonal components of variation across all transcripts, using the Basic Expression Workflow routine in JMP Genomics (SAS Institute, Cary, NC, USA). The contributions of the aforementioned biological covariates were assessed as a weighted sum of the proportion of each principal component (PC) explained. ANOVA was then used to assess whether clinical status impacts any of the major PCs individually. In addition, we assessed the correlation between each PC and white blood cell (available for 96% of subjects) and neutrophil counts (16% of subjects) as shown in Additional file 3. A summary of the workflow is provided in Additional file 4.

Since the loadings of the PC are only partially conserved between any two studies (including the phases) and there is no biological reason for axes of variance to be orthogonal, we also adopted an analysis of nine conserved axes of variation following the strategy we recently defined from re-analysis of multiple peripheral blood gene expression profiling datasets [17]. Each axis represents strong co-variance of several hundred transcripts with a correlation coefficient r > 0.7 that appear to represent different aspects of immune function. They are defined by an axis score that is generated as PC1 for a set of 10 blood informative transcripts that we have shown consistently correlate with the respective axis [17]. Each of these axis scores explains 70% to 95% of the variation in the set of 10 blood informative transcripts, compared with just approximately 25% for any randomly chosen set of 10 transcripts. Multiple regression of all nine axis scores on all of the transcripts was performed for each phase, and over 5,070 probes associated with at least one axis in either phase. Cross-matching of the list of significant associations between the two phases showed, on average, 81% overlap, ranging from 61% for axis 9 to 91% for axis 3. Those transcripts that are significantly associated at the approximate Bonferroni threshold of *P* < 10^-5^ in both phases (2,432 probes) were retained as axis-associated transcripts (Additional file 5).

### Statistical analyses

Differential gene expression between classes of subject (AMI versus non-AMI; cardiovascular death versus remainder; drug treatments) was evaluated by analysis of variance on the normalized data. Volcano plots [23] show the significance for each probe as the negative logarithm of the *P*-value (NLP) against the magnitude of difference (log 2 scale, 1 represents a 2-fold change).

For survival analysis, both cohorts were pooled and PC1 scores were categorized by outcome specific receiver operating characteristic (ROC) analyses using Youden’s index (Sensitivity - (1 - Specificity)) [26] to identify the threshold for 'high' and 'low' cardiovascular death-associated PC1 scores in both cohorts separately. The relationship between PC1 score and outcomes was determined using the Cox proportional-hazards regression in unadjusted models and in models adjusted for established risk factors that included age, gender, BMI, serum creatinine, diabetes, hypertension, hyperlipidemia, smoking, statin use, AMI and CAD (>50% luminal stenosis) all at baseline. The ability of the standard clinical model for predicting adverse events was calculated using the C-statistic before and after addition of the PC1 score.

Gene enrichment analysis was performed with the ToppGene Suite [27], and showed a highly significant enrichment (hypergeometric *P* = 4 × 10^-8^) for 18 genes annotated to the set of 222 genes known to be up-regulated in CD133^+^ relative to CD133^-^ hematopoietic stem cells in the 'Jaatinen_HSC_Dn' Molecular Signatures Database (MSigDB) [28] entry. No other significant multiple comparison-adjusted enrichments were reported.

## Results

Table 1 includes the demographic and clinical characteristics of subjects in the discovery phase (175) and replication phase (163). Of the 338 subjects, 65% were male, the mean age was 61 years, and 70% had significant CAD (>50% stenosis) and 18% were experiencing an AMI. Table 1 also contrasts the MI and non-AMI subjects, and shows that the AMI patients were more likely to be male, to have diabetes, significant CAD, and higher creatinine and Gensini scores as well as white blood cell and, where available, neutrophil counts (n = 55).

**Table 1:** Baseline characteristics

**Baseline characteristics**	**All subjects (N = 338)**	**Discovery cohort (N = 175)**	**Replication cohort (N = 163)**	**AMI (N = 60)**	**No AMI (N = 278)**	***P*-value**
Age (years)	62 ± 11	67 ± 10	56 ± 10	65 ± 11	61 ± 11	0.014
Male gender (%)	218 (65)	112 (64)	106 (65)	47 (78)	171 (62)	0.015
Caucasian race (%)	331 (98)	170 (97)	163 (100)	58 (97)	273 (99)	0.19
Systolic blood pressure (mmHg)	138 ± 23	142 ± 23	133 ± 21	135 ± 23	138 ± 23	0.36
BMI (kg/m^2^)	30 ± 7	29 ± 6	31 ± 8	29.6 ± 6.8	30.2 ± 7.0	0.53
Serum creatinine (mg/dl)	1.3 ± 1.1	1.3 ± 1.4	1.2 ± 0.7	1.6 ± 2.2	1.2 ± 0.7	0.009
Acute MI (%)	60 (18)	37 (21)	23 (14)	-	-	-
Old MI (%)	109 (32)	61 (35)	48 (30)	13 (22)	96 (35)	0.060
Current smoking (%)	73 (22)	34 (19)	39 (24)	8 (14)	65 (24)	0.09
Diabetes (%)	120 (36)	65 (37)	55 (34)	29 (48)	91 (33)	0.024
Hypertension (%)	258 (77)	142 (81)	116 (72)	46 (77)	212 (77)	0.98
Dyslipidemia (%)	263 (78)	133 (79)	102 (77)	47 (78)	216 (78)	0.95
Chronic heart failure (%)	76 (23)	41 (24)	35 (21)	15 (25)	61 (22)	0.61
LVEF (%)	54 ± 11	54 ± 11	54 ± 11	51 ± 11	54 ± 11	0.06
HDL (mg/dl)	43 ± 15	44 ± 14	41 ± 16	38 ± 14	44 ± 15	0.014
LDL (mg/dl)	92 ± 37	91 ± 36	94 ± 38	85 ± 33	94 ± 37	0.13
Triglyceride (mg/dl)	159 ± 115	150 ± 93	170 ± 137	154 ± 107	160 ± 117	0.73
Total cholesterol (mg/dl)	166 ± 45	164 ± 42	169 ± 48	153 ± 35	169 ± 47	0.02
History of CABG (%)	73 (21)	42 (24)	31 (19)	13 (22)	60 (22)	0.99
Gensini score	35 ± 54	38 ± 57	31 ± 55	48 ± 54	32 ± 54	0.046
Aspirin use (%)	83	131 (81)	131 (84)	48 (89)	214 (82)	0.2
Beta blocker use (%)	213 (67)	107 (67)	106 (67)	45 (82)	168 (64)	0.012
Clopidogrel use (%)	171 (55)	91 (56)	80 (53)	34 (64)	137 (53)	0.13
ACE-inh/ARB use (%)	192 (61)	100 (62)	92 (61)	35 (67)	157 (60)	0.35
Statin use (%)	244 (78)	129 (80)	115 (76)	44 (83)	200 (77)	0.33
>50% stenosis (in any major epicardial artery (%)	235 (70)	133 (76)	102 (64)	59 (93)	176 (64)	<0.001

*P*-value denotes differences between those with and without AMI. *Abbreviations: ACE-inh/ARB* angiotensin converting enzyme inhibitor or angiotensin receptor blockers, *BMI* body mass index, *CABG* coronary artery bypass graft, *HDL* high-density lipoprotein, *LDL* low-density lipoprotein, *LVEF* left ventricular ejection fraction, *MI* myocardial infarction.

### Differential expression associated with acute myocardial infarction

**Figure 1:**
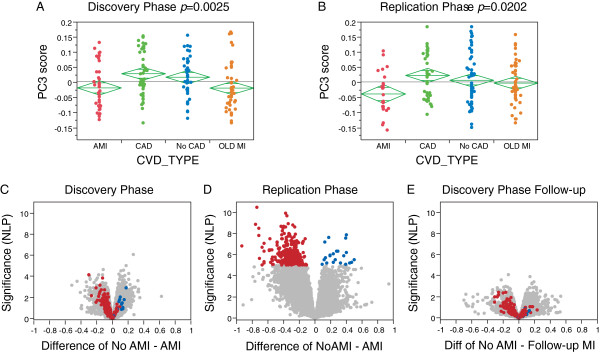
**Differential expression according to coronary artery disease status. (A,B)** PC3 scores by disease classification in the two phases of transcriptome profiling. *P*-values from ANOVA with three degrees of freedom for group effect, implying replicated differential expression between individuals presenting with AMI and those with NO CAD, CAD and OLD MI, which are not consistently divergent. **(C,D)** Volcano plots of significance against difference in expression in log2 units of SNM-normalized data with genes upregulated in patients with AMI to the left, and genes downregulated in AMI to the right. Colored transcripts are significant at *P* < 10^-5^ in phase 2. Almost all of these are differentially expressed in the same direction in phase 1, which, however, showed much less evidence for significant differential expression in the total sample (possibly reflecting lower technical quality, or unknown covariates biasing against the AMI association). **(E)** Volcano plot of differential expression between non-AMI individuals who have had an MI on follow-up (n = 11) and the remainder, all in phase 1.

Exploratory analyses indicated that as many as 4,500 transcripts may differ in abundance with respect to their CAD status, namely NO CAD, CAD, OLD MI, or AMI. The first five PCs explained 42% of the variation in the discovery phase and 46% in the replication phase. Notably, PC3 is significantly affected by CAD status in the same direction in both phases, with the AMI samples differentiated from the other three non-AMI samples (Figure 1A,B). The remaining NO CAD, CAD, and OLD MI groups are not significantly differentiated from one another. Similarly, there is no relationship between transcript abundance and angiographic burden of CAD as measured by the Gensini score. The absence of significant differential gene expression among the CAD and NO CAD groups was confirmed by ANOVA contrasting each of the three non-AMI classes (data not shown).

The differential expression in the AMI samples is shown in the volcano plots in Figure 1C,D. The effect of AMI is stronger in the replication phase, but the vast majority of the genes showing highly significant differential expression in either direction in the replication phase (red or blue spots highlighted in Figure 1D) showed the same direction of effect in the discovery phase (Figure 1C), indicating replication of the AMI effect. Remarkably, differential gene expression in 11 individuals who had an MI during the follow-up period for the discovery sample also tends to be in the same direction (Figure 1E; insufficient sample size for replication phase). Furthermore, Kiliszek *et al*. [13] reported 24 genes that were differentially expressed in a set of 28 patients experiencing AMI relative to controls, as well as at 6 months follow-up, and although these genes are not the most significant in our list, PC1 for their 24 genes is significantly associated with AMI in our cohort (one-tailed *t*-test, *P* = 5 × 10^-5^) and corresponds very closely to the major component of AMI-associated gene expression in our study. This differential ranking of top genes is to be expected as the most significant transcripts in any particular sample will be influenced by sampling variance.

**Figure 2:**
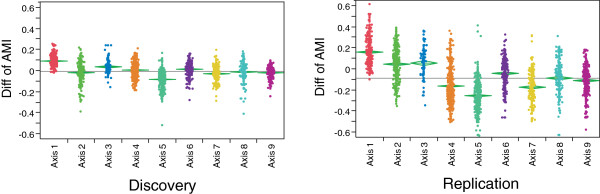
**Replicated association of axes of variation with myocardial infarction.** Each gene that is correlated with one of the nine axes in both phases is plotted, showing the mean difference between individuals with and without AMI (higher expression in AMI producing negative values). The overall relationship of the axes with AMI status is highly replicated, notably showing downregulation of axis 1 and upregulation of axis 5. The difference between the studies in axis 2 is readily explained because this axis is associated with BMI, which is elevated in the replication phase sample.

To explore the nature of the differential expression further, we evaluated the nine axes of variation that we recently showed are highly conserved in peripheral blood gene expression profiles from multiple studies in healthy and disease cohorts [17]. Each of 2,432 transcript probes associated with the same axis in both phases was classified as an axis gene. The directionality of the average difference in transcript abundance between the AMI and non-AMI samples for each gene, plotted by axis in Figure 2A,B, is highly concordant between the two phases. There is unambiguous downregulation of genes in axis 1 and upregulation of genes in axis 5 in AMI patients. In other studies, axis 1 is correlated with lymphocyte count and axis 5 with neutrophil count [17], and these relationships are confirmed for the subset of samples with cell counts in this study (Additional file 3). Neutrophil counts were not available for most individuals, but fitting white blood cells into a linear model absorbs the association of the axes with AMI (Additional file 6), indicating that much of the axis association is due to their correlation with white blood cell counts.

### eQTL analysis shows that differential expression in AMI involves changes in cell counts and gene expression

Evidence that the high expression of genes in axis 5 is only partially explained by neutrophilia is provided by cross-matching of the 1,987 genes associated with axis 5 at the 5% false discovery rate (FDR) with published gene expression profiles of distinct blood types from the Immunological Gene Consortium [29,30]. This indicates that 45% of 2,469 genes known to be neutrophil-enriched are associated with axis 5 in this study, and 55% of the axis 5 genes are neutrophil-enriched. The fact that the overlap is only partial suggests that neutrophil abundance is not the only explanation for the differential expression. Similarly, reduced lymphocyte abundance does not solely explain the low axis 1 scores in AMI patients, since only 44% of 1,746 axis 1 genes (at 5% FDR) are known to strongly associate with T-lymphocyte abundance. Furthermore, axes 8 and 9 are also enriched for genes involved in aspects of T-cell signaling, while axis 3 has an excess of genes annotated to B-cell signaling. None of these axes are associated with AMI. These results imply that differential gene expression in AMI patients is partly attributable to elevation of the ratio of neutrophils to lymphocytes, and partly to differential gene expression within these cell types.

To confirm this, we compared genotypic effects on gene expression between AMI and non-AMI samples in the discovery phase. A genome-wide association study measuring the effects of locally acting common polymorphisms on the abundance of each of the 14,343 transcripts was performed. Only the 153 Caucasian samples were included to prevent possible ethnicity-associated influences, and only SNPs with a minor allele frequency greater than 0.1 and located within 250 kb of the probe were assessed, also excluding probes known to contain SNPs to avoid hybridization artifacts [25]. Local genetic regulation of transcript abundance was detected for 355 probes representing 334 genes at *P* < 10^-5^, with a q-value FDR of 1%. On average, each lead eSNP (the strongest association at a locus) has a Pearson correlation with the associated transcript of 0.46, with a range from 0.27 to 0.90.

**Figure 3:**
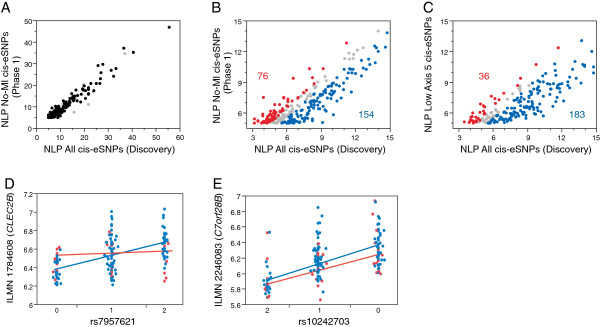
**eQTL analyses. (A-C)** Plots of significance (NLP) of local eSNPs with a transcript probe located within 250 kb of the SNP, contrasting the full Caucasian sample in the discovery phase with the same sample minus all individuals experiencing an MI. **(A)** The full range; **(B)** eSNPs in the range 5 < NLP < 15. Colored points differ between the full and non-AMI samples by at least 0.5 NLP units, red indicating higher significance in the smaller sample, and blue higher significance in the full sample. **(C)** The same analysis as **(B)** but missing 39 individuals with the highest axis 5 scores who were not experiencing an MI. Numbers show the number of eSNPs in the red and blue categories across the full range of NLP >3. **(D,E)** Representative plots of transcript abundance by genotype, colored with respect to MI status (red experiencing an MI, blue no-MI). Lines show the slope of the two classes, indicating an interaction effect in **(D)** but no interaction effect in **(E)**.

In order to evaluate whether AMI status influences local genetic effects on gene expression, we re-performed the eQTL analysis on just the 120 no-AMI Caucasian samples. This resulted in the reduction in significance of 154 associations (by at least 0.5 NLP value) as expected due to the reduced power in a smaller sample, but also led to the increase in significance of 76 associations, including detection of 45 novel associations, at *P* < 10^-5^ (Figure 3B). Additional file 2 lists the location, probe and SNP identity, and the Pearson correlation and NLP value for all 303 eSNPs detected at NLP >5 (*P* < 10^-5^) in the non- AMI sample, compared with the all Caucasian associations. Of the cases with increased genotype effects in the non-AMI sample, 72% show a significant interaction effect between genotype and AMI status. In each case they show a non-significant relationship between genotype and gene expression in the AMI samples where a highly significant one exists in the non-AMI samples (Figure 3D). By contrast, only 12% of the cases of reduced significance in the non-AMI samples showed an interaction effect, each with only a mild increase of AMI status on the genotypic effect (Figure 3E).

This result, that AMI reduces the genetic regulation of expression of a subset of genes, could simply be explained if the eSNP effect is predominately observed in lymphocytes and/or monocytes, so is diluted out in the AMI samples where neutrophils are more prevalent. To exclude this explanation, we removed an equivalent number of non-AMI high axis 5 samples from the total discovery phase dataset, and re-performed the eSNP evaluations. As expected, significance was reduced for 183 genes due to the loss of power, but only 36 cases of increased significance below the *P* < 10^-5^ threshold were observed. Consequently, the altered genotype effect is not simply a function of high axis 5 scores, but more often reflects an AMI-specific loss of influence on gene expression. To our knowledge, a study-specific disease-by-genotype interaction effect on local regulation of gene expression has only previously been reported for the effect of *Plasmodium* parasite load in infants with malaria in a study conducted in Benin [31].

### Gene expression signature of risk of cardiovascular death

In the combined cohort, there were 31 (9.5%) cardiovascular deaths during follow-up of 326 patients (12 patients, 3.6% were no longer available). In the discovery phase sample, analysis of variance contrasting 23 individuals experiencing cardiovascular death during follow-up with the remaining survivors revealed 244 probes in 238 genes that were significantly differentially expressed at *P* < 0.01. The first PC of these probes explains 23.3% of their variance in the joint sample of 338 individuals.

**Figure 4:**
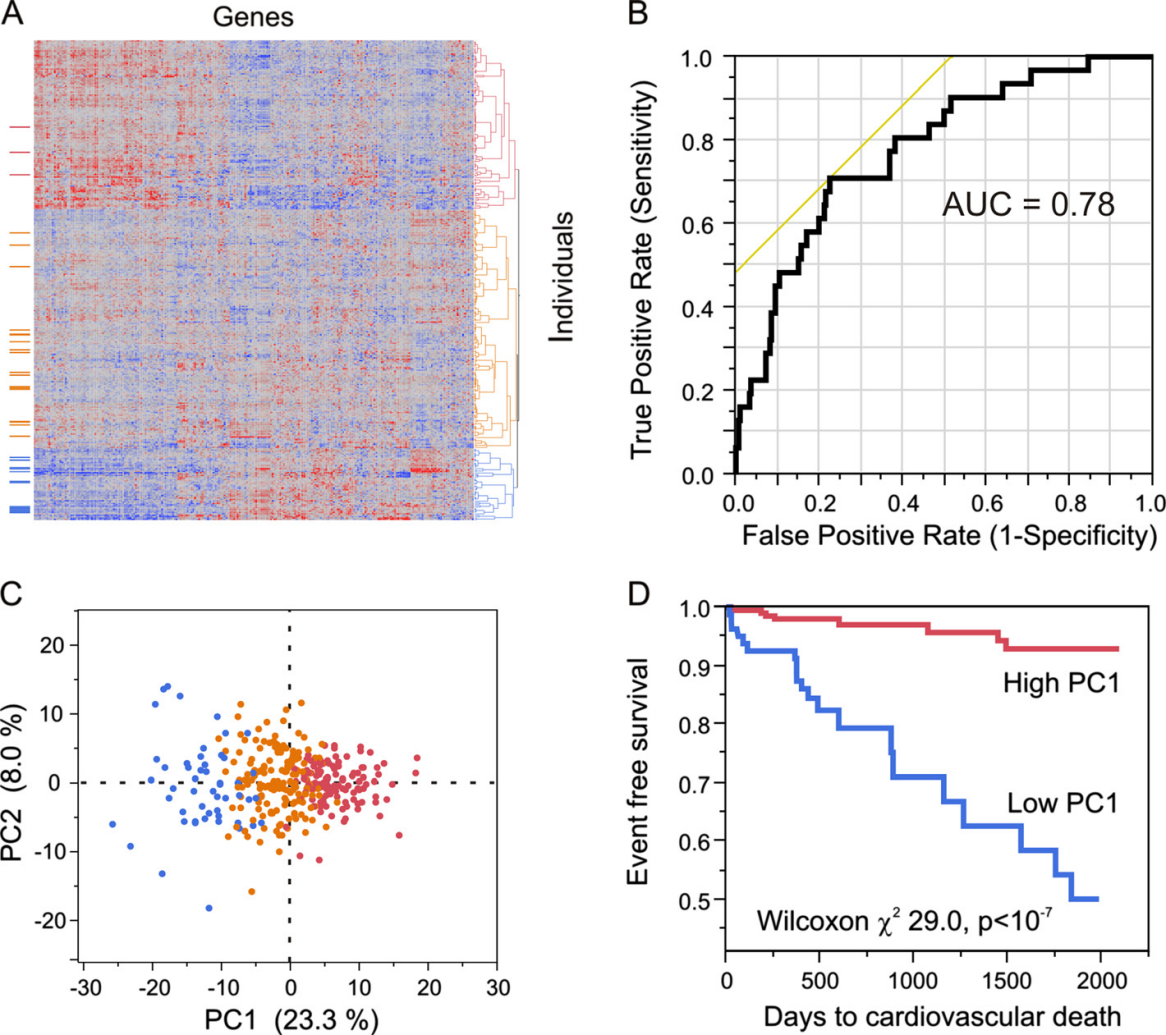
**Gene expression predictor of incident cardiovascular death. (A)** Two-way hierarchical plot of normalized transcript abundance (columns) by subjects (rows), highlighting the three deepest clusters. The column to the left illustrate subjects with cardiovascular death during follow-up: note the over-representation in the blue cluster and under-representation in the red one. **(B)** ROC for the sensitivity of PC1 of the 238 probe (230 gene) signature of cardiovascular death in the joint analysis. **(C)** Projection of hierarchical clusters on first two PCs. **(D)** Kaplan-Meier survival curve contrasting individuals in the bottom 29% of PC1 (blue curve; PC1 < -3.5), which includes 97 subjects (all 51 of the blue cluster and 46 of the orange cluster), and the remaining 71% (red curve; n = 240, PC1 > -3.5).

Two-way hierarchical clustering of these transcripts revealed three groups of individuals showing co-expression of these transcripts (Additional file 7). PC1 of these genes is predictive of cardiovascular death with an area under the ROC curve (AUC) of 0.80. This result was then verified in the replication phase sample in which eight patients experienced incident cardiovascular death, producing an AUC of 0.77. After combining the two datasets, hierarchical clustering recapitulates the clustering and a joint AUC of 0.78 was obtained (Figure 4B). Of the 219 subjects in the blue or orange clusters (Figure 4A), 28 (12.7%) died during follow-up compared with just 3 (2.5%) of the 119 subjects in the red cluster (2.5%), LRT χ^2^ = 17.8, *P* = 0.0006.

The best cutoff for the transcriptional PC1 score of the 238 differentially expressed genes in predicting cardiovascular death was determined to be -3.5 non-dimensional units using Youden’s index [26]. Kaplan-Meier survival analysis demonstrates a highly significant difference in survival between the 97 individuals with a PC1 score below -3.5 and the remaining 71% of subjects with PC1 score above -3.5 (*P* = <10^-7^; Figure 4D).

Univariate predictors of cardiovascular death indicated in Table 2 were age, low density lipoprotein, total cholesterol, serum creatinine, AMI, white blood cell count, presence of significant CAD >50%, left ventricular ejection fraction, and PC1 score, with hypertension trending towards significance. Multivariate cox proportional hazard analysis including univariate predictors with *P* < 0.20 reveals age, serum creatinine, and PC1 score < -3.5 as independent predictors of cardiovascular death during follow-up, where the hazard ratio for the PC1 score indicates a more than eight-fold increased risk of death. Furthermore, discrimination analysis shows that the addition of PC1 score to a baseline model comprising all of the terms listed in Table 2 was associated with improvement in the C-statistic (AUC 0.82 to 0.91, *P* = 0.03).

**Table 2:** Univariate and multivariate predictors of cardiovascular death

	**Cardiovascular death (N = 31)**			
	**Univariate**		**Multivariate**	
	**β**	***P*-value**	**HR**	***P*-value**
Age	0.05	**0.002**	1.07	**0.007**
BMI	-0.04	0.13	1.01	0.789
Male gender	-0.23	0.52	-	-
Diabetes	0.59	0.10	0.69	0.49
Hypertension	1.43	**0.05**	1.89	0.44
Smoking	0.39	0.33	-	-
Prior MI	-0.56	0.11	1.53	0.49
Statin use	-0.71	0.18	0.31	0.14
Aspirin use	0.19	0.66	-	-
LDL cholesterol	-0.01	**0.03**	1.002	0.91
Total cholesterol	-0.01	**0.01**	0.98	0.32
Serum creatinine	0.31	**<0.001**	1.28	**0.008**
Acute MI	0.48	0.29	-	-
White blood cell count	0.13	**0.01**	1.19	0.51
CAD >50%	0.97	**0.04**	2.68	0.15
LVEF	-0.03	**0.01**	0.96	0.08
PC1 score <3.5	2.08	**<0.001**	8.53	**<0.001**

Significant predictors are highlighted in bold. *Abbreviations: BMI* body mass index, *HR* hazard ratio, *LDL* low-density lipoprotein, *LVEF* left ventricular ejection fraction.

Additional file 8 lists the genes that are present in the signature. Diverse functions are represented, notably several genes involved in gylcerophospholipid metabolism and sphingomyelin-mediated signaling (*PRKCH*), thrombosis (*CD59*), leukocyte recruitment (*CD63*), splicing, and PI3K-Akt signaling, also with a highly significant enrichment for 18 genes known to be upregulated in CD133+ hematopoietic stem cells. Since half of these transcripts are positively and half negatively associated with PC1, it is unlikely that they simply report progenitor cell activity.

**Figure 5:**
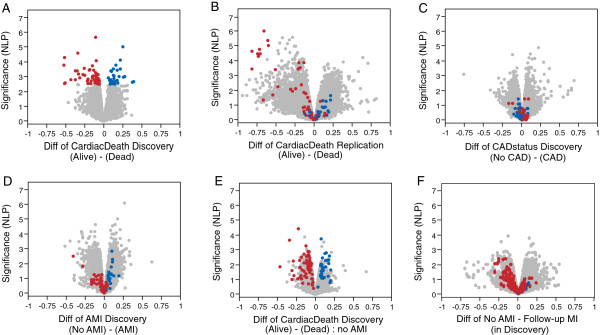
**Volcano plots contrasting differential expression with respect to CAD status, AMI, or incident cardiovascular death.** Data were transformed by the SNM procedure (see Additional file 9 for similar analysis of standard normalized data). **(A)** Cardiovascular death versus survivors in the discovery phase, where shaded points indicate transcripts that are differentially expressed at NLP >2.7 (5% FDR). **(B)** Replication of cardiovascular death direction of effect in the replication phase. **(C)** No CAD versus CAD, showing absence of overlap with AMI or cardiovascular death in direction of effects. **(D)** AMI versus no-AMI in the discovery phase, showing similarity of direction of differential expression relative to **(A)**. **(E)** Cardiovascular death versus survivors; same as **(A)** but after removing all individuals who were experiencing an AMI. **(F)** Individuals who experienced an AMI after enrolment versus not at all, also showing the cardiac death signature is in the same direction for predicting heart attack in general.

The differential expression associated with incident cardiovascular death appears to involve similar components of variation as the AMI-associated genes, since the PC1 score is correlated with those of axes 1 and 5. Even though the identities of most of the highly significant genes are distinct from the AMI-associated genes, 22 of the 230 PC1 genes are associated with axis 1 at *P* < 10^-5^. Figure 5 compares volcano plots between the two phases (Figure 5A,B), using the SNM normalization strategy (red and blue transcripts in all panels in Figure 5 are colored with respect to the cardiac death association in the discovery phase). The vast majority of genes upregulated in individuals in the discovery phase who subsequently died of a cardiovascular event are also upregulated in the replication phase individuals who have died, as well as in the patients experiencing an AMI (Figure 5D). Furthermore, when we remove the 37 patients from the discovery phase who were experiencing an AMI, and compute the association with subsequent death, these genes all remain upregulated (Figure 5E). Similarly, the downregulated genes in patients experiencing an AMI and who subsequently experienced cardiovascular death all strongly tend to co-vary in the same direction. As a negative control for these comparisons, Figure 5C shows that there is no bias of the cardiovascular death-associated genes with respect to CAD status. It is also noteworthy that the sign of differential expression in individuals who experienced a follow-up MI without death tends to be in the same direction as well (Figure 5F). Similar results using the linear regression normalization are shown in Additional file 9.

### Relationship between gene expression, subject characteristics and medication use

Ethnicity and BMI contribute to differences in transcript abundance (Additional file 1) as observed in our other cohorts [17,19]. We also assessed whether cardiovascular medications are associated with gene expression. One-way ANOVA on statins, angiotensin antagonists, beta blockers, clopidogrel, or aspirin usage did not demonstrate noteworthy drug-specific enrichment for gene expression. In either phase, we did observe significant association between axis scores and usage of clopidogrel or beta blockers, but these effects did not replicate in both phases (Table 2).

## Discussion

Our two novel findings relating to the relationship between gene expression variation and clinical syndromes associated with CAD are 1) demonstration of a clear transcriptomic signature of AMI that is indicative of a pro-inflammatory response, and 2) that a subset of this axis of peripheral blood gene expression is likely associated with the risk of cardiovascular death during medium-term follow-up [32]. Importantly, the PC1 gene score added predictive value to standard risk factors, with a hazard ratio over 8 in a multivariate model, and demonstrated improvement in the standard C-statistic. Moreover, our key results are based on replication in two independent phases of genomic data acquisition in the Emory Cardiovascular Biobank, and have been validated using two parallel modes of data normalization. Other findings are a lack of association between the transcriptome and the presence and severity of CAD, occurrence of previous MI, or with cardiovascular medication use.

### Gene expression associated with AMI and with incident cardiovascular death

We found an unambiguous association between a pro-inflammatory gene expression profile and presentation with AMI. The fact that samples were drawn within 2 to 24 hours after AMI, and that the two associated axes of variation (axis 5 and axis 1) are heavily enriched for upregulation of neutrophil and downregulation of T-lymphocyte gene activity, respectively, suggest that this pattern of gene expression may have been the result of AMI rather than its cause. However, these observations may not be entirely explained by neutrophilia that accompanies AMI [33,34]. First, not all neutrophil- and T-lymphocyte-enriched transcripts were altered to the same extent. Thus, in addition to modification of the ratio of cell types, there is differential activation of the co-regulated innate and adaptive gene functions. Secondly, 20% of the eQTL effects are stronger in subjects without AMI than in those with AMI, indicating altered genetic regulation of gene expression. Thus, we conclude that AMI is associated not just with an increase in neutrophil activity, but also with an alteration of gene activity in both neutrophils and lymphoctyes.Our results suggest that there may be a specific subset of leukocyte gene activity that is associated with a particularly high risk of death among individuals with CAD. Our current sample is too small to evaluate whether genotypic differences contribute to this risk category. We do not see any enrichment for eQTL in these genes, but the finding that genotypes affect response to a cardiac event also raises the question of whether enrichment with certain genotypes affects an individual’s physiological response to AMI. Just as with genome-wide association studies, it will likely take joint gene expression and whole genome genotype studies of tens of thousands of individuals with CAD to identify robust genomic biomarkers of risk for adverse events. This signature is, to some extent, co-regulated by whatever processes result in coordinate expression of genes in axis 1 and axis 5, but appears to involve additional coordinated regulation of transcription of a subset of genes with diverse molecular functions. Although the cardiovascular death signature appears to be related to that of AMI, it is only a subset of it, since comparison of Figure 5A,B with Figure 5D indicates that the transcripts most strongly associated with death in both phases are only weakly associated with AMI. Both the AMI and cardiovascular death associations are in the same direction in individuals who experienced a follow-up non-lethal MI, further indicating that there is some commonality to the cellular and transcriptional bases to the gene expression-based risk of adverse cardiovascular events.

In order for this profile to be incorporated into clinical practice, it would be favorable to ascertain a mechanism that explains the association between gene expression and risk of death. Possibilities include potential for plaque vulnerability, arrhythmogenesis, rapid occlusive thrombosis, mediation or repair of vessel walls, and regulation of signaling between the vascular endothelium and circulating blood cells. Understanding of the mechanism may lead to development of new therapeutic targets and subsequent interventions. The combination of traditional risk factors, genotypic risk from genome-wide association study variants [11,12], emerging biochemical predictors of cardiovascular death [15], and peripheral blood gene expression profiling nevertheless provides a framework for identifying a population at extraordinarily high risk for adverse outcomes, and for the aggressive management of cardiovascular disease that is personalized, predictive and participatory, with the hope that it may also lead to preventive risk reduction [35].

### Lack of gene expression association with coronary artery disease

Several peripheral blood microarray studies have reported transcriptomic signatures associated with the presence of underlying CAD. The CardioDx Corus®CAD signature [9,10], which was identified in a sample of over 1,000 individuals, was not replicated in our cohort. This 23 gene quantitative RT-PCR assay is reported to improve the predictive accuracy of traditional risk factors for atherosclerosis in a sex-specific manner in non-diabetics and was validated in the multicenter PREDICT trial. It includes genes that capture activity in a small number of biochemical pathways and/or represent the abundance of neutrophils. The most likely reason for lack of our ability to replicate their findings is confounding clinical attributes, given that the majority of our cohort already have CAD and are receiving diverse pharmacological treatments, as a result of which the control healthy adult sample is relatively small.

We also failed to observe a strong relationship between gene expression and the use of aspirin, statins, angiotensin antagonists, beta blockers, or clopidogrel. Although clopidogrel showed a significant association for several hundred genes in the discovery phase, these did not replicate in the replication phase. It is likely that the very high covariance of gene expression into axes of coordinated gene expression that explain over half of the variance among individuals readily induces correlations that do not replicate. These axes are correlated with genetic, environmental and unknown sub-clinical measures in a multivariate manner, and may have high false positive rates in relatively small samples. These considerations underscore the relevance of the observed replication of the cardiovascular death and AMI associations.

Nevertheless, the association of the 238 transcript signature with cardiovascular death needs to be replicated in an independent prospective cohort because only 31 of the 338 subjects reported here have suffered a cardiovascular death. A strength of our study is that all of our findings replicate in two phases, and we have performed careful normalization using two parallel modes of analysis to confirm the results and relate them to our prior evaluation of the axes of variation in human peripheral blood. Future research will need to carefully measure complete blood counts, as these were not available for the AMI patients in this study and the confounding of neutrophilia with the inflammatory and cardiovascular death signatures was inferred from a subset of the data and other studies. Longitudinal analyses of gene expression will also assist in interpretation of the effect of AMI on future risk, and possibly also evaluation of whether lifestyle changes that reduce CAD risk also alter the peripheral gene expression profiles in a favorable direction.

## Conclusion

A signature of gene expression related to inflammation and altered T-cell signaling correlates with incident AMI and a subset of these associate with risk of cardiovascular death. The core result evaluated across both phases of the study produces a combined AUC of 0.78 and improves the C-statistic of risk classification when evaluated alongside 12 clinical measures. If independently validated, a peripheral blood transcript abundance test has potential, in conjunction with measures of cholesterol, serum creatinine, and white blood cell counts, to generate a significant predictor of risk of cardiovascular death in patients with CAD. The signature appears to be independent of the influence of conventional CAD medications. We envisage a tiered approach to genomic medicine for CAD risk assessment that incorporates traditional risk factors, biochemical biomarkers, genotypic risk scores, and gene expression.

## Abbreviations

AMI: acute myocardial infarction; AUC: area under the ROC curve; BMI: body mass index; CAD: coronary artery disease; eQTL: expression quantitative trait locus; FDR: false discovery rate; MI: myocardial infarction; NLP: negative logarithm of the *P*-value; PC: principal component; PCA: principal component analysis; ROC: receiver operating characteristic; SNM: Supervised Normalization of Microarray; SNP: single nucleotide polymorphism.

## Competing interests

The authors declare that they have no competing interests.

## Authors’ contributions

GG and AQ designed the study and supervised the genomics and clinical aspects, respectively; NG and DE were involved in data acquisition and integration of cardiovascular parameters; JK and GG carried out the statistical and genomic analyses with advice from NC and JS; NG and AQ performed the survival analysis and risk assessment.

## Authors’ information

Joint senior authors are Arshed A Quyyumi and Greg Gibson.

## Supplementary Material

Additional file 1: Figure S1 Variance components of gene expression. The histograms show the weighted average contribution of each variance component to the variation in the first five principal components of SNM normalized gene expression in the discovery and replication phases. BMI3 is a three-level categorization of BMI (obese, BMI >30, over-weight 25 < BMI < 30, normal, BMI <25). CAD status is the four levels described in the text. Plate effects were removed in the SNM model. Residual is unexplained variance. Click here for file

Additional file 2: Table S1 List of eSNPs at NLP >5 comparing Caucasian and no AMI. Click here for file

Additional file 3: Table S2 Association of gene expression with cell counts. Click here for file

Additional file 4: Figure S2 Schematic of the gene expression profiling analysis. Click here for file

Additional file 5: Table S3 List of probes associated with axes. Click here for file

Additional file 6: Table S4 Association of white blood cells with axes. Click here for file

Additional file 7: Figure S3 Survival analysis in both phases. **(A, B)** Hierarchical clustering was performed independently for the discovery **(A)** and replication **(B)** phases with the 238 probe (230 gene) signature of cardiovascular death. Red, blue and orange clusters are as in Figure 4, and horizontal notches beside each heat map show individuals who have died due to a cardiovascular event during the follow-up period. **(C, D)** Corresponding ROC curves for PC1 as a function of cardiovascular death in both phases independently. Note that **(B)** and **(D)** use the same genes in the replication phase that were discovered in the first phase. Click here for file

Additional file 8: Table S5 List of genes associated with cardiovascular disease. Click here for file

Additional file 9: Figure S4 Volcano plots by alternate normalization. Volcano plots as in Figure 5, but following linear modeling to remove technical effects (RNA quality and batch) from z-scores. The plots are narrower than with the SNM normalization because this mode of normalization also equilibrates the variance, which makes the analysis at the level of relative rank of gene expression rather than fold-change. Nevertheless, the key results are concordant between the two strategies. Click here for file
