# Supplementary figures and images for: Gene expression profiles associated with acute myocardial infarction and risk of cardiovascular death
Source: Genome Med. 2014 May 30;6(5):40. doi: 10.1186/gm560 (PMC4071233; doi:10.1186/gm560)

**Additional File 1 (Figure 1).** Kim *et al*, 2014

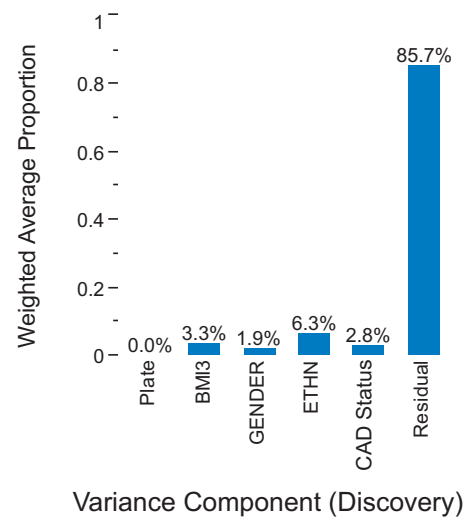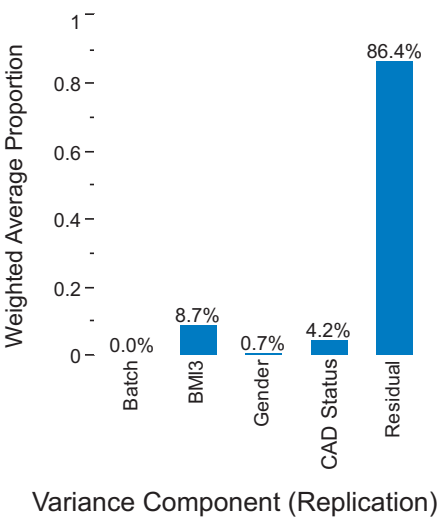

Supplement: Additional file 1: Figure S1 — Variance components of gene expression. The histograms show the weighted average contribution of each variance component to the variation in the first five principal components of SNM normalized gene expression in the discovery and replication phases. BMI3 is a three-level categorization of BMI (obese, BMI >30, over-weight 25 < BMI < 30, normal, BMI <25). CAD status is the four levels described in the text. Plate effects were removed in the SNM model. Residual is unexplained variance. [file gm560-S1.pdf]

**Additional File 4 (Figure 2).** Kim *et al*, 2014

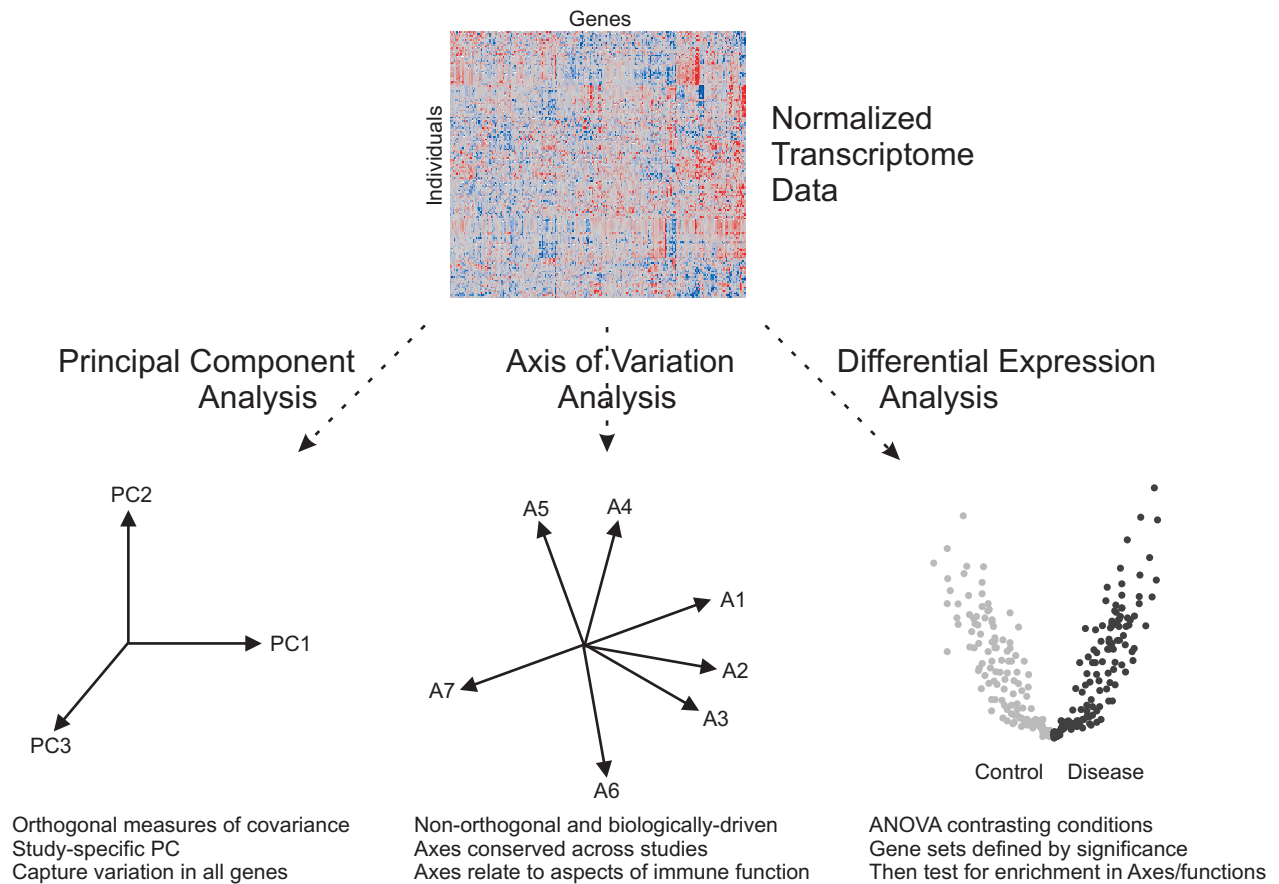

Supplement: Additional file 4: Figure S2 — Schematic of the gene expression profiling analysis. [file gm560-S4.pdf]

**Additional File 7 (Figure 3 Kim *et al*, 2014).**

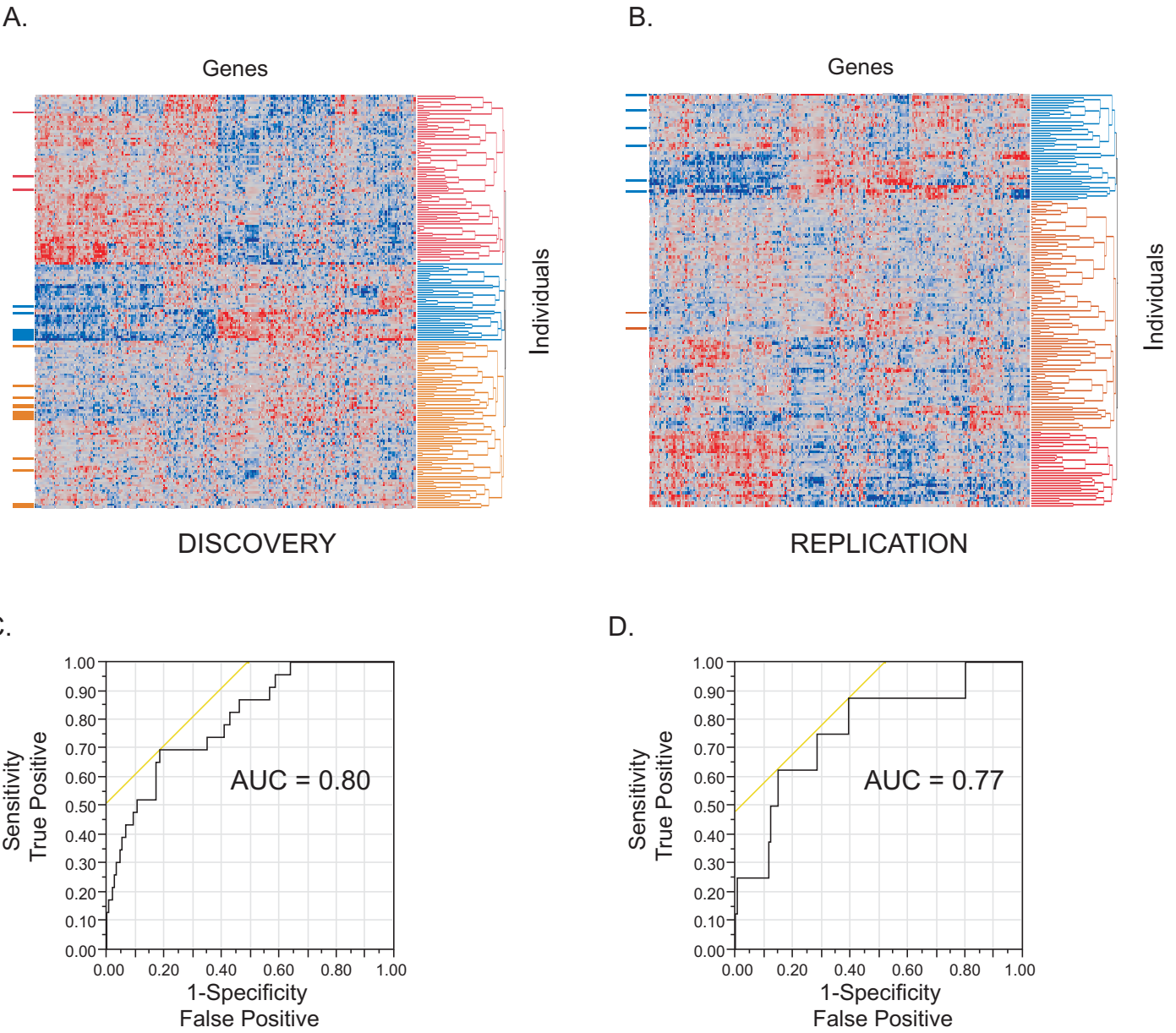

Supplement: Additional file 7: Figure S3 — Survival analysis in both phases. (A, B) Hierarchical clustering was performed independently for the discovery (A) and replication (B) phases with the 238 probe (230 gene) signature of cardiovascular death. Red, blue and orange clusters are as in Figure 4, and horizontal notches beside each heat map show individuals who have died due to a cardiovascular event during the follow-up period. (C, D) Corresponding ROC curves for PC1 as a function of cardiovascular death in both phases independently. Note that (B) and (D) use the same genes in the replication phase that were discovered in the first phase. [file gm560-S7.pdf]

**Additional File 9 (Figure 4).** Kim *et al*, 2014

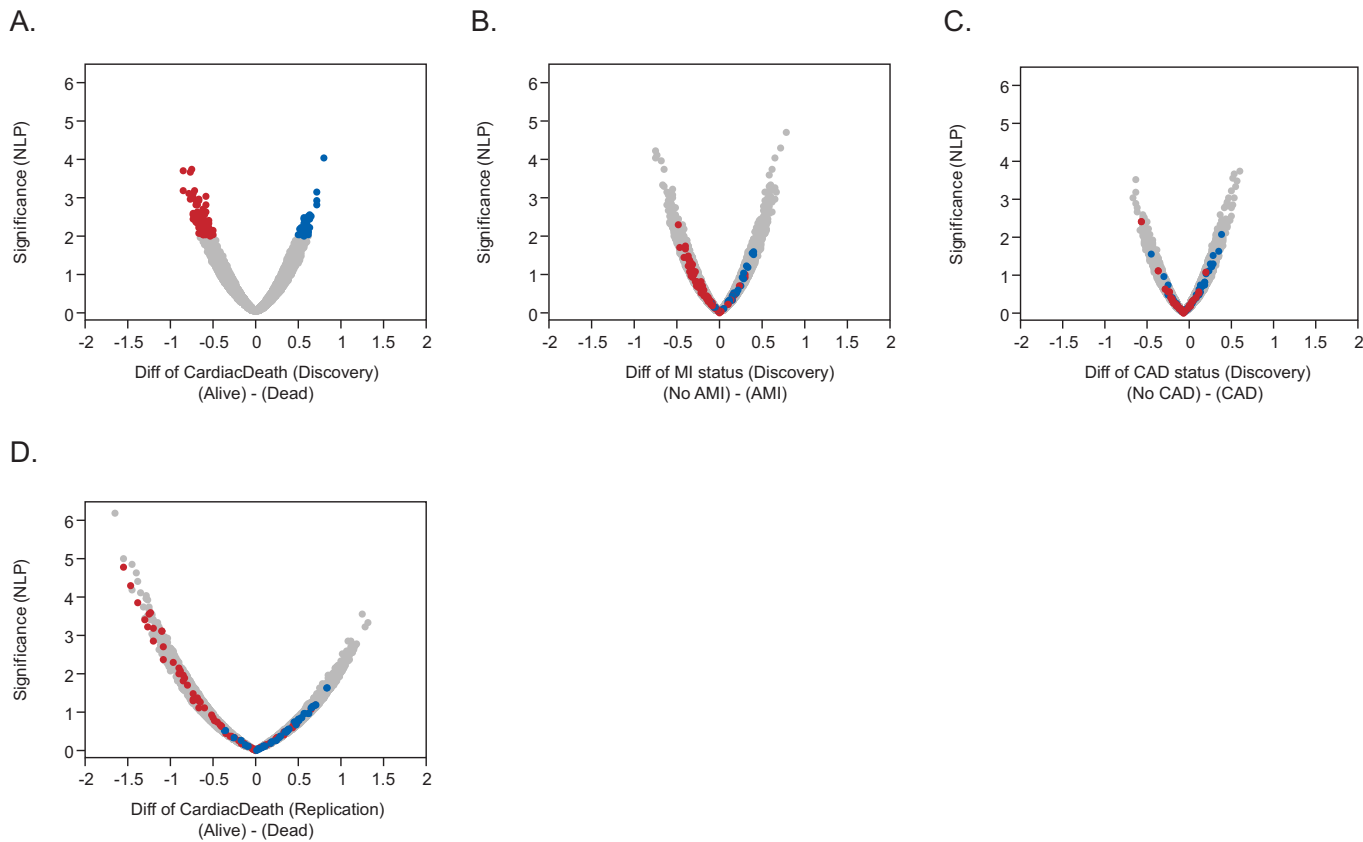

Supplement: Additional file 9: Figure S4 — Volcano plots by alternate normalization. Volcano plots as in Figure 5, but following linear modeling to remove technical effects (RNA quality and batch) from z-scores. The plots are narrower than with the SNM normalization because this mode of normalization also equilibrates the variance, which makes the analysis at the level of relative rank of gene expression rather than fold-change. Nevertheless, the key results are concordant between the two strategies. [file gm560-S9.pdf]
