# Supplementary material for: Gene expression profiles associated with acute myocardial infarction and risk of cardiovascular death
Source: Genome Med. 2014 May 30;6(5):40. doi: 10.1186/gm560 (PMC4071233; doi:10.1186/gm560)
Supplement: Additional file 3: Table S2 — Association of gene expression with cell counts. [file gm560-S3.pdf]

**Additional File 3 (Table 2)** Association of Gene Expression Components with Cell Counts

| Variable | WBC | Neutrophils |
|---|---|---|
| PC 1 (16.0%) | $2.8\times10^{-4}$ | 0.18 |
| PC 2 (11.2%) | $9.5\times10^{-5}$ | 0.25 |
| PC 3 (6.2%) | $4.1\times10^{-7}$ | 0.21 |
| PC 4 (3.5%) | 0.002 | 0.02 |
| PC 5 (3.0%) | 0.37 | 0.98 |
| Axis1 | $6.9\times10^{-11}$ | 0.02 |
| Axis2 | 0.16 | 0.96 |
| Axis3 | 0.13 | 0.25 |
| Axis4 | $7.7\times10^{-4}$ | 0.46 |
| Axis5 | $4.7\times10^{-11}$ | 0.006 |
| Axis6 | 0.19 | 0.05 |
| Axis7 | 0.095 | 0.003 |
| Axis8 | 0.003 | 0.44 |
| Axis9 | $4.7\times10^{-8}$ | 0.25 |
| Death_joint_PC1 | $3.9\times10^{-10}$ | 0.06 |

Table shows p-values for regression of white blood cell (WBC) or Neutrophil count on the indicated Prinicpal Component (with amount of variance it captures) or Axis of Variation score.
