# Supplementary material for: Gene expression profiles associated with acute myocardial infarction and risk of cardiovascular death
Source: Genome Med. 2014 May 30;6(5):40. doi: 10.1186/gm560 (PMC4071233; doi:10.1186/gm560)
Supplement: Additional file 5: Table S3 — List of probes associated with axes. [file gm560-S5.pdf]

Additional File 5 (Table 4) Covariance of WBC, Axis 1 and Axis 5 with Acute MI

	Estimate	Chi-square	P-value	AUC
WBC	-0.26	22.9	5×10^{-6}	0.71
Axis 1	0.21	14.6	1×10^{-4}	0.66
Axis 5	-0.16	7.9	0.0049	0.65
WBC with Axis 1	-0.23 / 0.09	16.3 / 2.2	5×10^{-5} / ns	0.74
WBC with Axis 5	-0.25 / -0.04	18.0 / 0.5	2×10^{-5} / ns	0.72

Estimates for the bottom two rows are for the WBC and Axis score respectively When both terms are included in the covariance model, indicating that the WBC Axis associations are largely due to their correlation with WBC.
